# Cardiac contractile function and mitochondrial respiration in diabetes-related mouse models

**DOI:** 10.1186/s12933-014-0118-7

**Published:** 2014-08-21

**Authors:** Camille Marciniak, Xavier Marechal, David Montaigne, Remi Neviere, Steve Lancel

**Affiliations:** EA 4484 – Physiology Department, Faculty of Medicine, Lille 2 University, 1, place de Verdun, Lille, 59045 France

**Keywords:** Metabolic syndrome, Diabetes, Heart, Mitochondria, Respiration, Streptozotocin, High-fat diet

## Abstract

**Background:**

Pathophysiological processes underlying diabetic-related cardiomyopathies are complex. Mitochondria dysfunction is often described as a cause of cardiac impairment but its extent may depend on the type of experimental diabetes. Here we proposed to compare drug- or diet-induced models of diabetes in terms of metabolic features, cardiac and mitochondrial functions.

**Methods:**

Mice were fed with regular chow or fat-enriched diet. After three weeks, they received either citrate or streptozotocin injections for five consecutive days. Metabolic parameters, myocardial contractile function and mitochondrial respiration were measured after three more weeks. Fat mass volumes were assessed by magnetic resonance imaging. Oral glucose tolerance test, insulin tolerance test, triglyceride and adipocytokine quantification were evaluated to establish metabolic profiles. Cardiac function was assessed *ex vivo* onto a Langendorff column. Isolated cardiac mitochondria respiration was obtained using high-resolution oxygraphy.

**Results:**

Mice fed with the fat-enriched regimen presented abdominal obesity, increased blood glucose, elevated leptin level, glucose intolerance, and insulin resistance. Mice treated with streptozotocin, independently of the regimen, lost their capacity to release insulin in response to glucose ingestion. Mice fed with regular chow diet and injected with streptozotocin developed cardiac dysfunction without mitochondrial respiration defect. However, both groups of high-fat diet fed mice developed cardiac alterations associated with reduction in mitochondrial oxygen consumption, despite an increase in mitochondrial biogenesis signalling.

**Conclusions:**

We explored three animal models mimicking type 1 and 2 diabetes. While cardiac dysfunction was present in the three groups of mice, mitochondrial respiration impairment was only obvious in models reproducing features of type 2 diabetes.

According to the American Diabetes Association, diabetes affects more than 25 million people in the United States and will impinge 366 million people in the world in 2030 [1]. Despite established compounds or drugs in development [2] and surgical interventions [3], human and economical costs remain enormous. Development of new experimental models reproducing diabetes mellitus and its complications is indispensable to have a deeper understanding of the pathophysiology and to develop innovative therapeutics.

Several species are used to reproduce diabetes such as nonhuman primates [4] or pigs [5]. The most frequently used animals are rodents, especially mice, because of their cost, size and availability. On the first hand, spontaneous monogenic modified mice such as db/db and ob/ob mice [6,7] as well as animals fed with high-fat diet have been developed. They share common features of type 2 diabetes, such as glucose intolerance, insulin resistance and dyslipidemia. On the other hand, OVE26 and Akita mice or injection of the toxic pancreatic agent streptozotocin (STZ) have been documented to reproduce characteristics of type 1 diabetes [8-10]. In both cases, cardiovascular dysfunction has been described either in basal conditions or under biological stress like β-adrenergic stimulation [11].

Among the numerous hypotheses advanced to explain diabetes-related cardiomyopathy, including calcium mishandling, sarcomere disruption or oxidative stress [12], mitochondrial dysfunction has been described as one of the most important. Indeed, mitochondria occupy approximately 30% of the heart volume [13] and these organelles are constantly solicited to produce ATP, which is required for cardiac contraction. It is thus obvious that defects in mitochondrial population quality may impair cardiac function. Many murine models of type 2 diabetes, including mice with genetic modifications, fed with high-fat diet associated or not with low doses of STZ, extensively described an association between cardiac dysfunction and mitochondria alterations, including reduced ATP production, defects in mitochondrial respiration despite stimulation of biogenesis signalling and modified proteome [14-16]. Such observations have also been obtained in the human heart from type 2 diabetic patients [17]. Surprisingly, cardiac mitochondrial dysfunction in models of type 1 diabetes is less characterized, differs from type 2 diabetes and from a model to another [10,18,19]. Finally, because of the variety of protocols published in distinct papers, it is not clear whether the intensity of mitochondrial dysfunction is correlated with the severity of cardiac dysfunction and whether mitochondrial impairment occurs at the same time in both types of diabetes.

In this study, we evaluated cardiac function and respiration of heart mitochondria in three different models of diabetes. First, we compared metabolic parameters such as glucose tolerance or insulin resistance in mice fed with regular chow diet and injected with STZ, mice fed with high-fat diet treated with citrate buffer or STZ to mice fed with normal diet injected with citrate buffer. Then, we measured myocardial contractile function and mitochondrial respiration in order to determine whether mitochondrial alterations precede diabetic-related cardiomyopathy.

## Methods

### Experimental models

Five-week old female C57/BL6J mice (Charles River, L’Arbresle, France) were randomly separated in two groups: the first one had free access to normal chow diet (ND), the other one to high-fat diet (HFD, D12492, SSNIFF, Soest, Germany). Food composition and energy sources are provided Table 1. Then, both groups of mice received either streptozotocin (60 mg/kg intraperitoneally, 1 injection per day for five consecutive days, Sigma Aldrich, St Quentin Fallavier, France), or the equivalent volume of citrate buffer that was used to dissolve streptozotocin (0.1 M, pH 4.5, 1 injection per day for five days). After injections, mice were housed for three more weeks and fed *ad libitum* with their respective food. Thus, after six weeks of feeding, four groups were obtained: ND/CITRATE, ND/STZ, HFD/CITRATE and HFD/STZ. There were at least two full days between tests to avoid potential stress confounding effects. All experimental procedures were approved by our institutional guidelines (DDSV Permit Number 59–350206) and were conducted according to NIH instructions.

**Table 1:** **Nutrient composition and energy sources of normal and high-fat diets**

	**Normal diet**	**High-fat diet**
Crude protein (%)	16.4	24.1
Fat (%)	4	34.6
Carbohydrate (%)	48.5	25.3
Fiber (%)	18.5	6
Ash (%)	4.9	6
Energy density (kJ/g)	12.6	24
Calories from proteins (%)	22	19
Calories from fat (%)	12	60
Calories from carbohydrates (%)	66	21

### Magnetic resonance imaging of fat distribution

Once anesthetized by inhaling isoflurane, mice were imaged on a Biospec 7-Tesla magnetic resonance imaging unit (Biospec, BrukerBioSpin SA, Wissembourg, France). Sequences were acquired in the abdomen, around the kidney region. The T1 spin-echo images were obtained with a thickness of 1 mm, a repetition time of 200 ms and an echo time of 27 ms. Data were analyzed with OsiriX software. Subcutaneous and abdominal fat mass volumes (expressed in cm^3^) were determined manually.

### Oral glucose tolerance test OGTT

In order to evaluate glucose tolerance, mice were subjected to a 12-h fasting period. Then, they were force-fed with a glucose solution (2 mg/g total body weight) in approximately 200 μL of water. One drop of blood obtained from a tail incision was used to determine glucose concentration by the use of a glucose meter. Measurements were obtained before ingestion and 10, 20, 30, 60 and 120 min after glucose challenge.

### Insulin tolerance test ITT

Fasted mice received intraperitoneally fast insulin (0.5 mU/g total body weight in saline solution). Blood glucose concentration was measured before and 15, 30 and 60 min after the injection.

### Blood collection and analysis of plasma

After fasting, tail incision was made and blood (100 μL) was collected into EDTA-coated tubes. Sample was spun at 1,000 *g* for 15 min and plasma was aliquoted and stored at −80°C until use. Plasmatic adiponectin, leptin, TNF-α, IL-6 and MCP1 were quantified with multiplex immunoassay kits (Millipore, Molsheim, France). True triglycerides were measured with the determination kit from Sigma Aldrich (St Quentin Fallavier, France), according to manufacturer’s instructions.

### Insulin response to glucose challenge

Blood samples (30 μL) were collected from fasted mice. Then, animals were force-fed with a glucose solution (2 mg/g total body weight). Ten minutes later, 30 μL of blood were withdrawn for plasma preparation. Then, insulin was measured with the insulin (Mouse) EIA kit (Alpco, Salem, NH, USA) according to manufacturer’s instructions.

### Pancreas immunohistochemistry

After mouse euthanasia, pancreas was dissected, fixed in Bouin solution and embedded in paraffin. Then, sections of 6 μm thickness were prepared and put onto superfrost slides. After paraffin removal, tissue was rehydrated in solutions of decreasing alcohol concentration. Slides were immersed into citrate buffer (0.01 M, pH 6) in order to unmask antigens. Then, endogenous peroxidase activity was blocked by incubating sections in 3% H_2_O_2_ diluted in methanol. After incubation in PBS + 3% bovine serum albumin, monoclonal anti-insulin antibody (1/100, AbD Serotec Colmar, France) was added to the sections and incubated at 4°C overnight. After rinsing, peroxidase-conjugated anti-mouse antibody was incubated for 1 h at room temperature. 3,3′-diaminobenzidine (Bethyl Laboratories, Inc., Montgomery, TX, USA) was applied for 15 min at room temperature. Slides were counterstained by immersion in hematoxylin. After rinsing, slides were mounted and observed under microscope with × 100 and × 400 magnification.

### Isolated and perfused heart

After cervical dislocation, heart was excised and aorta was cannulated onto a Langendorff column. Heart was perfused in a retrograde manner with oxygenated (O_2_ 95%, CO_2_ 5%) modified Krebs-Henseleit (consisting in NaCl 120 mM, KCl 4.8 mM, KH_2_PO_4_ 1.2 mM, MgSO_4_ 1.2 mM, NaHCO_3_ 25 mM, CaCl_2_ 1.25 mM, glucose 11 mM) at 37°C with a constant perfusion rate (2.5 mL/min). In some experiments, 0.8 μg/mL isoprenaline was added in the buffer. A metal hook was inserted into the apex. The hook was connected to a calibrated dynamometer, which was connected to a pre-amplifier linked to the acquisition station (Powerlab, ADInstrument, Oxford, United Kingdom). Heart spontaneously beat with a 2 g preload. Developed force and its first derivatives were measured in absence or presence of isoprenaline. Heart work was defined as the product of force x heart frequency.

### Mitochondria isolation and function

Cardiac mitochondria were isolated as previously described [20]. Mitochondria (200 μg) were introduced into O2K oxygraph chambers (Oroboros Instruments, Innsbruck, Austria) to evaluate their oxygen consumption in presence of different compounds. In a first series of experiments, pyruvate (5 mM), glutamate (5 mM) and malate (2 mM) were added. After signal stabilization, ADP (0.5 mM) was injected in order to measure oxygen consumption when oxidative phosphorylation occurs. The second series of experiments consisted in adding palmitoylcarnitine (20 μM) in presence of malate (2 mM). Then ADP (0.5 mM) was injected into the chambers in order to evaluate respiration with substrates subjected to β-oxidation.

### Mitochondrial DNA copy number

Total DNAs were extracted with QIAamp DNA mini kit (Qiagen, Courtaboeuf, France) according to manufacturer’s instructions. Nucleic acid concentration and purity were measured at 260 and 280 nm with the Nanodrop reader (Nanodrop products, Wilmington, DE, USA). Then 40 ng of DNA were used to perform quantitative PCR with FastStart Universal SYBR Green (Roche Applied Science, Meylan, France). The following primers were utilized: mtCOII Forward AACCATAGGGCACCAATGATAC, Reverse GGATGGCATCAGTTTTAAGTCC [GeneID: 17709]; PPIA Forward ACACGCCATAATGGCACTGG, Reverse CAGTCTTGGCAGTGCAGAT [GeneID: 268373].

### RTqPCR

Total RNAs were extracted from 20 mg heart tissue with 1 mL Trizol reagent and purified with PureLink Micro-to-Midi kit (Life Technologies SAS, Saint Aubin, France). DNA was systematically removed by DNase treatment. Then, reverse transcription with random hexamers and Transcriptor First Strand cDNA Synthesis kit (Roche Applied Science) was performed according to manufacturer’s instructions. Amplification was done with FastStart Universal SYBR Green and the following primers: PGC-1α Forward CGGAAATCATATCCAACCAG, Reverse TGAGGACCGCTAGCAAGTTTG [NM_008904], 18S Forward CGGCGACGACCCATTCGAAC, Reverse GAATCGAACCCTGATTCCCCGTC [NR_003278].

### Statistical analysis

Presented data are means ± SEM. Analysis was performed with GraphPad Prism software 5.0 (San Diego, CA, USA). Statistical analysis was carried out using two-tailed unpaired *t*-test when comparing two groups and one-way ANOVA followed by Bonferroni post-hoc tests when comparing more than three groups. Two-way ANOVA was performed to determine time influence on the measured parameters and was used for analysis of food intake, calorie intake, variation of weight, OGTT and ITT.

## Results

### Food intake, weight gain and fat distribution

**Figure 1:**
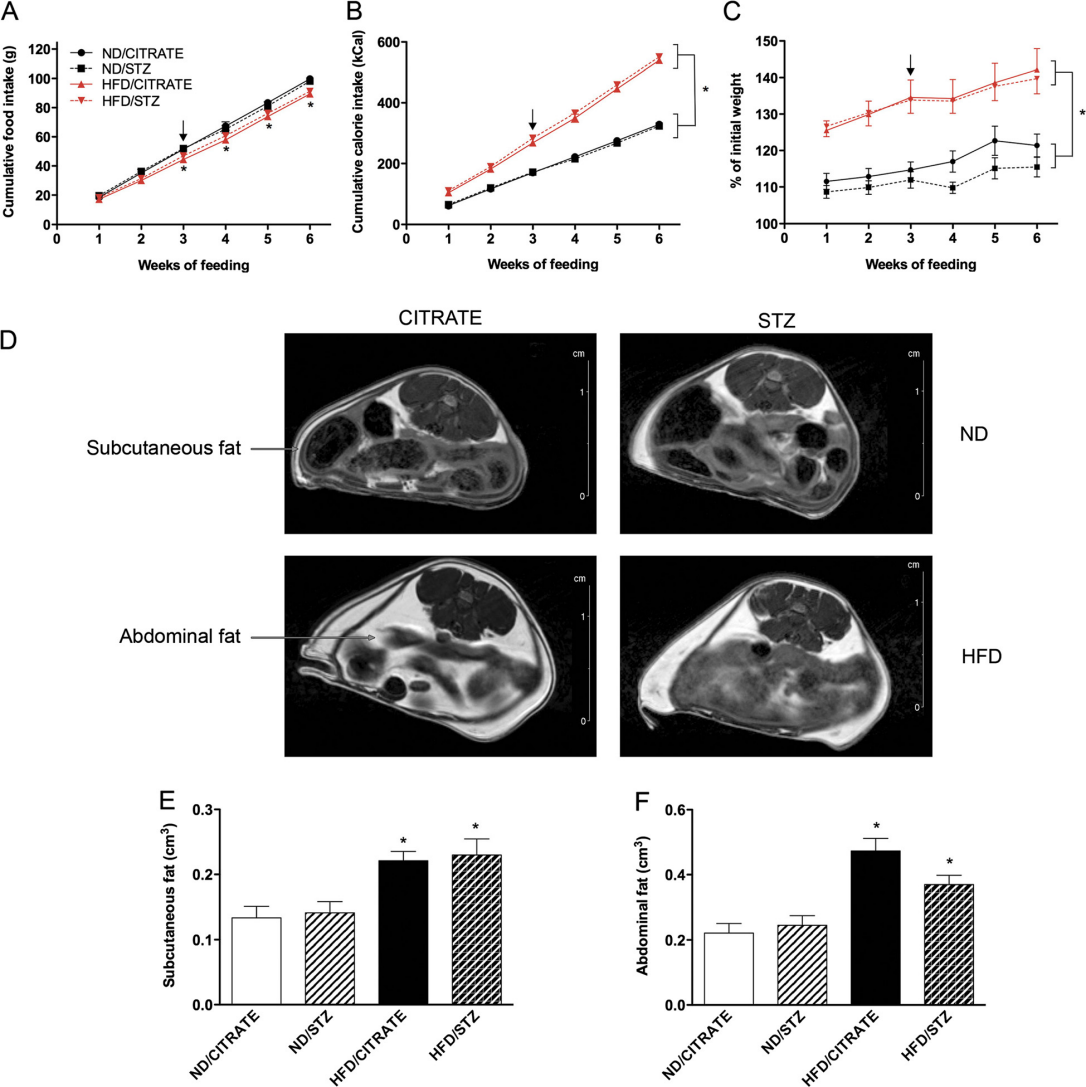
**Food intake, weight gain and fat volume in the four groups of mice. (A)** Cumulative food intake and **(B)** cumulative calorie intake over weeks of feeding. **(C)** Gain of weight compared to initial weight. Circle: ND/CITRATE, square: ND/STZ, triangle: HFD/CITRATE, diamond: HFD/STZ mice. Arrows indicate when streptozotocin (STZ) or citrate buffer were injected. **(D)** MRI images of abdominal region in ND/CITRATE, ND/STZ, HFD/CITRATE, HFD/STZ mice fed for 6 weeks. Arrows indicate subcutaneous and abdominal fat. Volume quantification of **(E)** subcutaneous and **(F)** abdominal fat. n = 8 per group, *p < 0.05 vs. ND/CITRATE mice.

Prior to cardiovascular and mitochondrial evaluations, we compared metabolic phenotypes of ND/STZ, HFD/CITRATE and HFD/STZ to ND/CITRATE. Three weeks after the beginning of the regimen, mice fed with HFD started eating less than their littermates under ND (Figure 1A). Despite this difference, cumulative energy intake in the HFD mice was significantly higher than in the ND animals from the first to the last week of specific diet (Figure 1B). Mouse weight increased faster in HFD groups than in the ND ones (Figure 1C). Injections of STZ (60 mg/kg total body weight) did not modify the amount of ingested food as ND/STZ and HFD/STZ ate as much as ND/CITRATE and HFD/CITRATE, respectively (Figures 1A and B). Similarly, body weights between citrate- or STZ-injected mice within ND or HFD groups were not significantly different (Figure 1C).

To further characterize their phenotypes, mice were subjected to MRI to quantify fat mass. As depicted in Figure 1D, fat distribution between citrate- and STZ-injected ND mice was similar. These results were confirmed by image quantification (Figures 1E and F). In both groups of HFD mice, hypersignal was stronger in subcutaneous and abdominal compartments (Figure 1D). Analysis revealed that, compared to ND/CITRATE, subcutaneous fat volume increased by 77% while abdominal fat volume doubled (Figures 1E and F) in both HFD groups. STZ injections had no effects on fat deposition neither in ND nor in HFD mice (Figures 1D-F).

### Glucose intolerance

**Figure 2:**
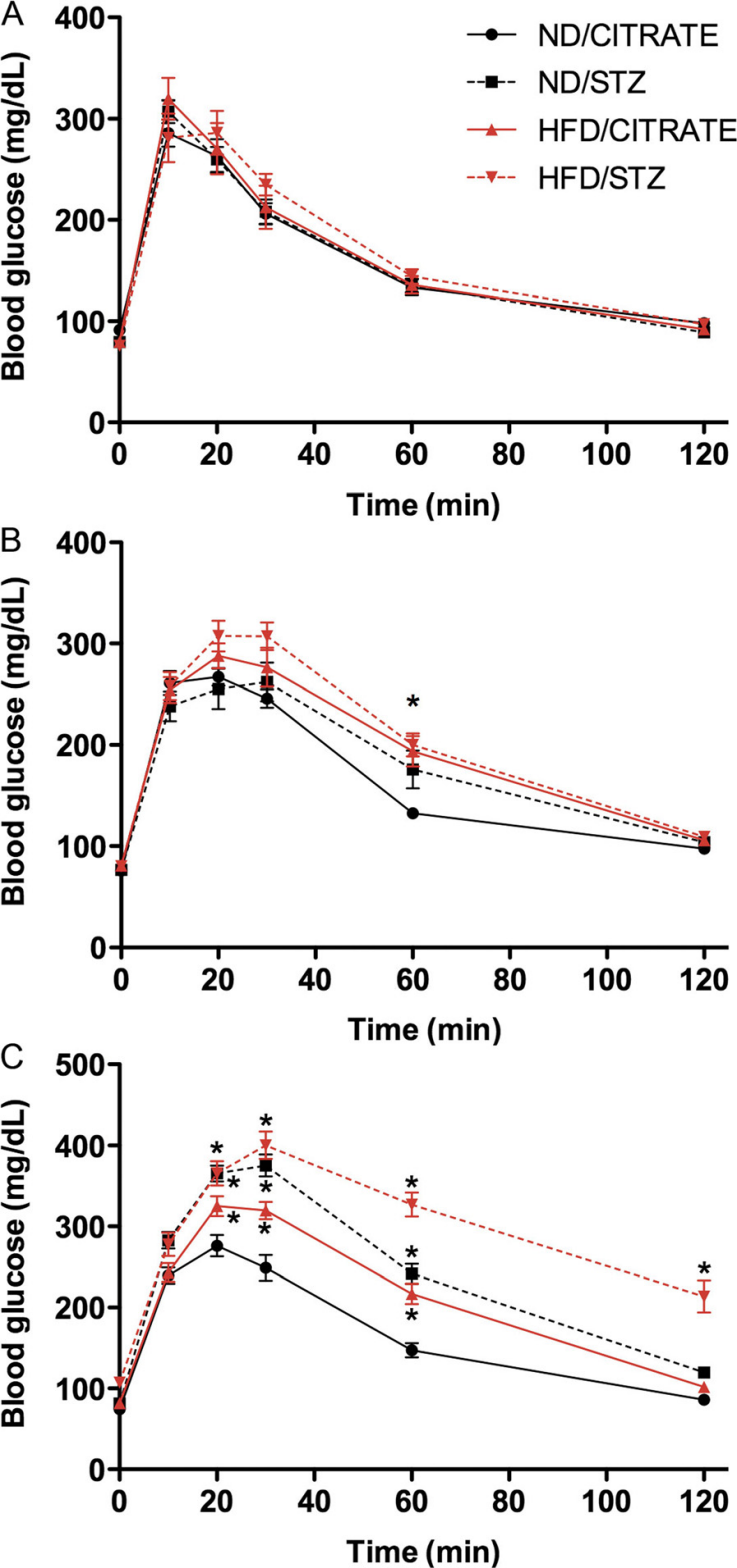
**Oral glucose tolerance tests of mice fed for one, three and six weeks.** After glucose ingestion, blood glucose concentration was measured at 0, 10, 20, 30, 60, 120 min later in mice fed for one **(A)**, three **(B)** or six **(C)** weeks. Circle: ND/CITRATE, square: ND/STZ, triangle: HFD/CITRATE, diamond: HFD/STZ mice. n = 16 per group, *p < 0.05 vs. ND/CITRATE.

OGTT was performed to evaluate glucose tolerance in the four different groups either after one week of feeding (no injections), three weeks (just before injections of citrate or STZ), or six weeks, i.e. three weeks after the first injections. As presented Figure 2A, mice had similar response to glucose ingestion after one week of HFD with no injections. After three weeks of feeding (Figure 2B), there were no significant differences between ND mice that will subsequently be injected with citrate buffer (ND/CITRATE) or STZ (ND/STZ). Similar results were obtained within the HFD groups (Figure 2B). Nevertheless, as soon as three weeks under specific regimens, both groups of mice fed with HFD started showing glucose intolerance, as their blood glucose concentration remained elevated 60 min after ingestion compared to ND (Figure 2B). Finally, OGTT was performed three weeks after citrate or STZ injections (Figure 2C). Compared to ND/CITRATE mice, ND/STZ and mice fed with HFD that received or not STZ had higher glucose concentration 20, 30 and 60 min after glucose force-feeding. Plus, after two hours, glucose concentration remained above 200 mg/dL in the HFD/STZ group (Figure 2C). All subsequent experiments were performed six weeks after the onset of the regimen.

### Insulin resistance and secretion capacity

**Figure 3:**
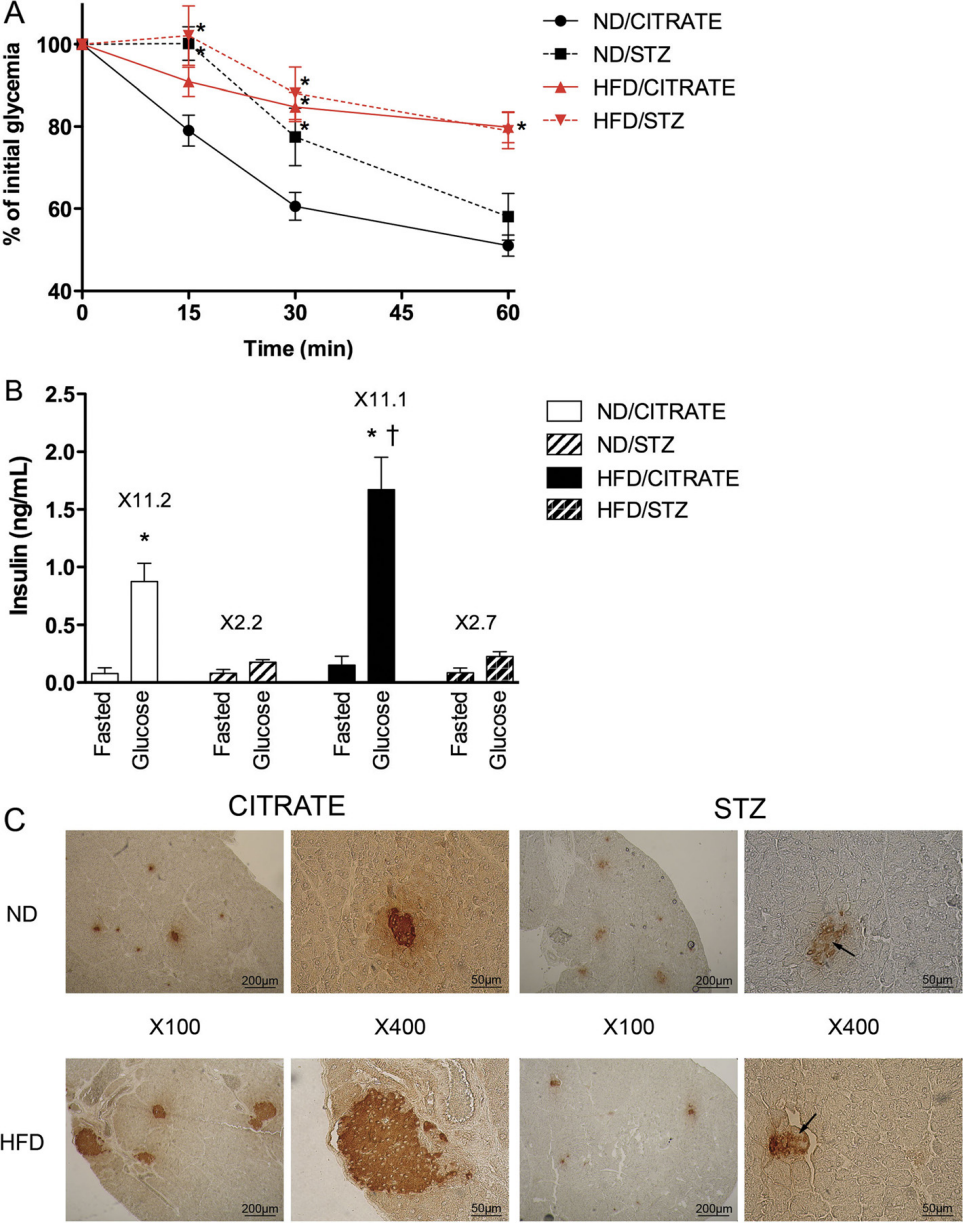
**Streptozotocin and high-fat diet induced insulin abnormalities after six weeks of feeding. (A)** Insulin tolerance test. Blood glucose was measured 15, 30 and 60 min after insulin injection. n = 10, * p < 0.05 vs. ND/CITRATE. **(B)** Insulin secretion in response of glucose ingestion. n = 5, *p < 0.05 compared to fasted conditions, † p < 0.05 HFD/CITRATE vs. ND/CITRATE. **(C)** Immunohistochemistry of pancreas using an anti-insulin antibody. Arrows indicate cells with lower staining intensity. Magnifications were ×100 and ×400, as indicated.

In HFD and HFD/STZ groups, slope of glucose concentration reduction after insulin injection was reduced compared to ND/CITRATE mice (Figure 3A). In addition, after 60 min, blood glucose concentration was reduced by only 20% in HFD-STZ and HFD/CITRATE while it decreased by 50% in both ND/CITRATE and ND/STZ (Figure 3A). ND/STZ mice had significant higher glucose concentrations 15 and 30 min after insulin injection.

We then evaluated glucose-stimulated insulin release by measuring circulating insulin before and after an oral glucose challenge. As illustrated in Figure 3B, insulin levels underwent a 11-fold increase either in ND/CITRATE or HFD/CITRATE mice. Mice treated with STZ lost their capacity to respond to glucose as insulin levels barely increased by 3 (Figure 3B). Moreover HFD/CITRATE mice significantly released more insulin than ND/CITRATE littermates (Figure 3B).

Finally, we qualitatively evaluated whether such alterations could be attributed to reduced size of pancreatic islets and insulin content, as STZ is a known-pancreatic poison targeting β-cells. Islet staining intensity seemed reduced in ND/STZ and HFD/STZ compared to ND/CITRATE mice (Figure 3C). In HFD/CITRATE group, islet size appeared bigger than in ND/CITRATE group but the staining intensity was not affected (Figure 3C). As expected, size of islets was reduced in both groups treated with STZ (Figure 3C).

### Metabolic parameters

Blood glucose concentration in fasted animals was significantly higher, although moderate, in ND/STZ, HFD/CITRATE and HFD/STZ as compared to ND/CITRATE (Table 2). Triglyceride and leptin levels mildly augmented in mice fed with HFD, independently of STZ treatment (Table 2). Inversely, compared to ND/CITRATE, adiponectin level was divided by 2 in HFD/CITRATE group while it diminished by 30% in HFD/STZ animals (Table 2). ND/STZ mice had equivalent amount of adiponectin compared to ND/CITRATE mice (Table 2). Regarding the pro-inflammatory status, neither MCP-1 (not detectable), nor TNF-α, nor IL-6 was significantly increased although a trend could be observed in ND/STZ, HFD/CITRATE and HFD/STZ compared to ND/CITRATE animals (Table 2).

**Table 2:** **Blood parameters after six weeks of diet**

	**ND/CITRATE**	**ND/STZ**	**HFD/CITRATE**	**HFD/STZ**
Glucose (mg/dL)	74 ± 2	84 ± 3 *	83 ± 2 *	112 ± 6 *
True triglycerides (mg/dL)	52 ± 4	60 ± 10	90 ± 8 *	103 ± 11 *
Leptin (ng/mL)	1.0 ± 0.2	1.1 ± 0.1	1.9 ± 0.2 *	2.3 ± 0.3 *
Adiponectin (μg/mL)	30.3 ± 3.1	30.3 ± 6.1	15.6 ± 0.9 *	21.4 ± 1.5 *
TNF-α (pg/mL)	3.8 ± 0.5	6.0 ± 1.2	5.4 ± 0.8	4.5 ± 0.4
IL-6 (pg/mL)	7.5 ± 0.9	12.4 ± 2.0	14.7 ± 1.8	13.0 ± 2.8

Data are means ± SEM. n = 8–10 per group, *p < 0.05.

### Cardiac function

**Figure 4:**
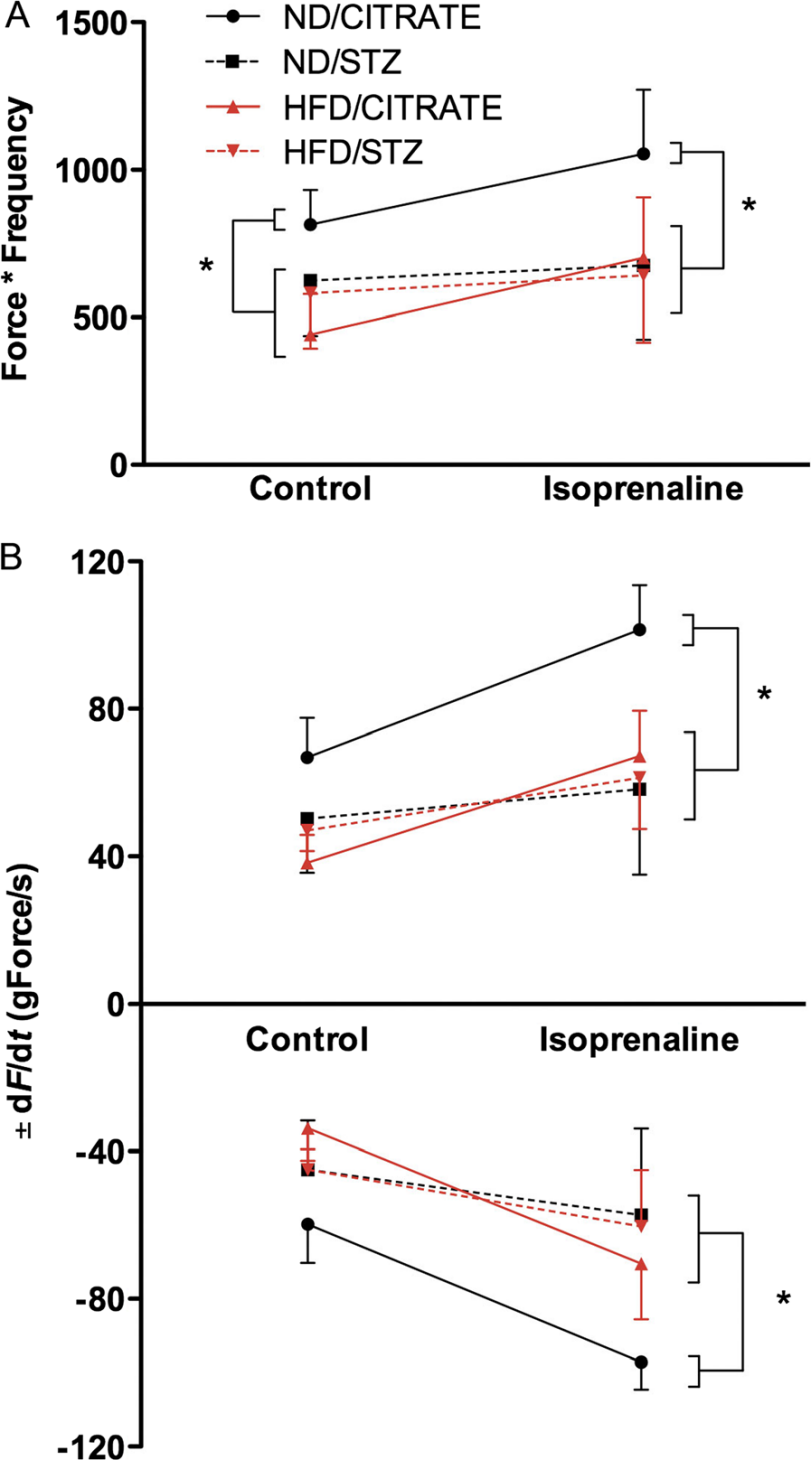
**Cardiac function alterations in diabetic mice. (A)** Spontaneous heart work measured *ex vivo* and **(B)** force first derivatives +/− d*F*/d*t* in presence or in absence of isoprenaline. Circle: ND/CITRATE, square: ND/STZ, triangle: HFD/CITRATE, diamond: HFD/STZ, n = 4–5 per group, *p < 0.05 vs. ND/CITRATE.

Isolated heart function was assessed onto a Langendorff apparatus. Under basal conditions, heart work (Figure 4A) and first derivatives + d*F*/d*t* and -d*F*/d*t* (Figure 4B), index of contractility and relaxation respectively, were reduced in ND/STZ, HFD/CITRATE and HFD/STZ compared to ND/CITRATE. In presence of isoprenaline, a β-adrenergic receptor agonist, both ND/CITRATE and HFD/CITRATE hearts increased cardiac work (Figure 4A) and first derivatives (Figure 4B). In contrast, ND/STZ and HFD/STZ hearts did not significantly respond to β-adrenergic stimulation (Figure 4).

### Mitochondrial respiration and biogenesis

**Figure 5:**
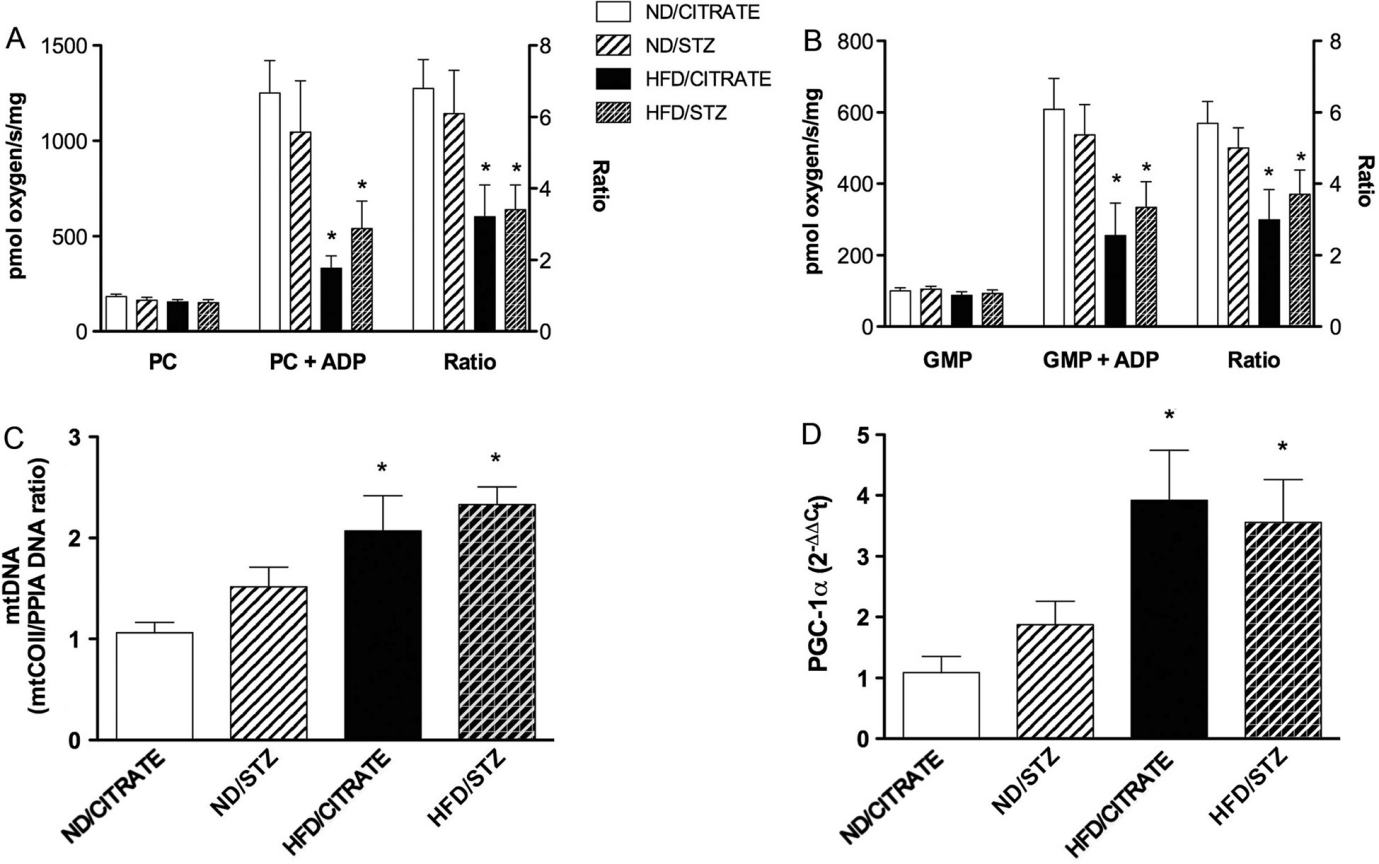
**Mitochondrial parameters after six weeks of diet.** Isolated mitochondria respiration in presence of **(A)** palmitoylcarnitine/malate or **(B)** glutamate/malate/pyruvate in absence (left) and in presence (middle) of ADP. Respiration ratio refers to right Y axis. **(C)** Mitochondrial DNA relatively to genomic DNA. **(D)** Expression of Pgc-1α mRNA in the four different groups after six weeks. n = 5, *p < 0.05 vs. ND/CITRATE.

Mitochondria were isolated from heart and analyzed with an oxygraph. When mitochondria were incubated in presence of palmitoylcarnitine and malate, respiration rate was similar in the four groups. Addition of ADP in the respiration media led to a 6.8- and 6.1-fold increase in oxygen consumption in the ND/CITRATE and ND/STZ mice, respectively (Figure 5A). This increase in respiration was significantly lower in HFD/CITRATE and HFD/STZ mice (Figure 5A). Similarly, no differences between groups were observed in presence of glutamate/malate alone (Figure 5B). Activation of oxidative phosphorylation by ADP resulted in a 5-fold increase in ND/CITRATE and ND/STZ whereas it increased by 3 in the HFD/CITRATE and HFD/STZ (Figure 5B). These mitochondrial alterations observed in HFD/CITRATE and HFD/STZ were associated with an increase in mitochondrial biogenesis signalling. Indeed, compared to ND/CITRATE, mtDNA copy number was higher in HFD/CITRATE and HFD/STZ (Figure 5C). Similar results were obtained for PGC-1α mRNA expression (Figure 5D).

## Discussion

The aim of this study was to characterize the metabolic phenotype of three diabetes-related animal models induced by HFD and the pancreatic poison STZ and to evaluate whether cardiac dysfunction was always associated with mitochondrial respiration impairment.

As a prerequisite before assessment of myocardial and mitochondrial functions, we evaluated metabolic features of the three diabetes-related models obtained in C57BL6/J mice, a strain known to respond to diet-induced obesity and to develop insulin resistance and glucose intolerance [21]. First, HFD mice gained more weight and consequently developed both subcutaneous and abdominal adipose tissues, as assessed by MRI. This was associated with a mild plasma true triglyceride elevation. Visceral adipose tissue, now considered as an endocrine tissue [22], produces pro-inflammatory cytokines such as TNF-α and IL-6 [23]. Here, we could not detect significant elevation in these two molecules. This may be attributed to the relatively short-term regimen (6 weeks) or to the C57BL6/J genetic background. Indeed Balb/c or FVB/N mice are more prone to develop HFD-induced inflammation [21]. Abdominal adipocytes also secrete leptin and adiponectin. As expected, the elevation in visceral fat was associated with increase in leptin circulating levels while adiponectin was reduced. Despite higher leptin concentration in the HFD fed animals, food intake barely reduced and mice kept accumulating fat, consistently with other studies [24]. STZ had no effects on leptin and adiponectin levels.

A deeper analysis of diabetes-related features consisted in studying glucose intolerance and insulin resistance. HFD/CITRATE or HFD/STZ mice had a lower rate of glucose disposal after insulin injection and glucose ingestion, as well as a moderate increase in fasting blood glucose concentration, featuring an insulin resistant status. Several mediators such as leptin, which reduces insulin-induced fatty acid oxidation and triacylglycerol synthesis [25,26], or fatty acids, which compete with glucose for their mitochondrial oxidation [27,28], are commonly suggested as contributors of insulin resistance and glucose intolerance. As observed in other experimental studies and in humans [29-31], we also detected bigger pancreatic β-cell islets and higher plasmatic insulin levels ten minutes after glucose ingestion in HFD/CITRATE mice. This metabolic phenotype indicates that HFD/CITRATE mice manifested features of a pre-diabetic state. We reproduced effects of STZ, an alkylating compound causing beta pancreatic cell death, described by others [32-34]. Indeed, ND/STZ and HFD/STZ mice released less insulin consecutively to glucose stimulation. Taken together, these results suggest that ND/STZ, HFD/CITRATE and HFD/STZ were more related to type 1 diabetes, pre-diabetes and established type 2 diabetes, respectively.

Diabetes-related cardiovascular alterations have been documented in both type 1 and type 2 rodent models [16,35-38]. Here we aimed to determine whether intrinsic myocardial contractile function was affected in these different settings of diabetes. In order to avoid confounding effects of circulating leptin, triglycerides, cytokines or insulin on the heart, we used isolated and perfused heart preparations onto a Langendorff apparatus. First, isolated hearts from STZ-treated animals did not respond to β-adrenergic stimulation. This could be related to a reduction in β1 and β2 adrenoreceptor expression while the β3 isoform, mediating negative inotropic effect, may rise [39]. Then, despite the mild elevation in fasting blood glucose and triglyceride concentration and an exploration close to the onset of diabetes, we found a marked intrinsic contractile dysfunction of the myocardium in the three diabetes-related models. Increased fatty acid oxidation at the expense of glucose utilization, expansion of connective tissue, oxidative stress, calcium mishandling and reduction in SERCA expression have been proposed to explain diabetic cardiac dysfunction [12,35]. In addition, several studies identified mitochondria as a potential cause of myocardial dysfunction in diabetes. Thanks to the early development of diabetic cardiomyopathy, we decided to explore whether this organ dysfunction was always associated with mitochondrial impairment.

We highlighted a profound mitochondrial respiration reduction in insulin-resistant HFD/CITRATE and HFD/STZ mice. Consistently studies on cardiac-specific knock-out mice for the insulin receptor (CIRKO) demonstrated that lack of insulin signalling in the myocardium leads to impaired contractile function along with mitochondria respiratory defects and ATP synthesis rate reduction that progress with age [40]. In addition, reduction of mitochondrial respiration was associated with stimulation of mitochondrial biogenesis signalling as evidenced by increased PGC-1α mRNA expression and mtDNA copy number. PGC-1α is a major transcription factor controlling mitochondrial biogenesis. Genetic deletion of PGC-1α causes reduced cardiac ATP production and the incapacity to respond to a stress such as exercise or adrenergic stimulation [41,42]. On the contrary, Lehman et al. [43] showed that, despite PGC-1α-mediated mitochondria number increase, contractile function was severely affected because of sarcomere misalignment. These studies emphasize that mitochondrial function had to be tightly regulated to exert beneficial effects on cardiac function. Mitochondrial population quality also depends on autophagy and dynamics, processes that are also involved in diabetes. For instance, in a severe model of type 1 diabetes, Xu et al. [44] described that autophagy is reduced in the diabetic heart. Surprisingly, inhibition of autophagy exerted cardioprotective effects, probably through at least in part mitophagy activation. Alterations of mitochondrial dynamics have also been reported in failing diabetic human hearts [45]. Although we did not examined oxidative damage in these hearts, increased in mitochondrial reactive oxygen species production may also contribute to the mitochondrial phenotype. Nakamura et al. [46] demonstrated that, for instance, in db/db mice, mitochondrial-derived ROS generation due to hyperglycemia and lipid accumulation activated p53 and its target SCO2. As a consequence, higher expression of SCO2 resulted in increases in fatty acid uptake, mitochondrial oxygen consumption and ROS production.

In the present study, we did not observe defects in mitochondrial respiration in ND/STZ mice, neither with pyruvate/glutamate nor with palmitoylcarnitine as substrates, while cardiac dysfunction was patent, suggesting that defects in mitochondrial respiration *per se* may not be the primary cause of cardiac dysfunction. While studies described similar results in rats [47], others reported that palmitoylcarnitine-dependent mitochondrial respiration could remain identical between control and STZ-injected despite a reduction in ATP production rate [48]. More recently, in a chronic model of type 1 diabetes, Vadvalkar et al. [49] demonstrated that heart mitochondria may have substrate inflexibility as their respiration was reduced only with non-fatty acid substrates. They also showed that protein lysine acetylation reproduced inhibition of non-fatty acid oxidation by mitochondria. The authors thus hypothesized that discrete mitochondrial changes such as protein lysine acetylation could impact oxidative phosphorylation before the onset of type 1 diabetic cardiomyopathy. However, in our study, we could not detect mitochondrial impairment with pyruvate/glutamate. This may be explained by the early investigation after type1-like diabetes induction as well as the mild phenotype we observed in terms of hyperglycemia and dyslipidemia. This model may not recapitulate the whole pathogenesis of a more severe and chronic type 1 diabetes. Finally, other mitochondrial functions may be affected during diabetes. Indeed a recent report [50] identified that ATP-dependent potassium channel expression is diminished by type 1 diabetes. As a consequence, diazoxide-induced depolarization was lower in diabetic mitochondria. Interestingly, interfibrillar mitochondria were more sensitive to diazoxide than subsarcolemmal mitochondria. This result underlines that mitochondrial populations may be differently affected by diabetes.

In conclusion, mechanisms responsible for the diabetic-related cardiomyopathies depend on duration, type and severity of diabetes. Injections of streptozotocin in mice fed with regular chow diet led to a type-1 like diabetic mouse model that displayed cardiovascular alterations without mitochondrial respiration impairment. On the contrary, cardiac dysfunction of mice fed with HFD that received or not STZ was associated with a dramatic reduction in mitochondrial respiration despite mitochondrial biogenesis activation.

## Abbreviations

ADP: Adenosine diphosphate
ANOVA: Analysis of variance
DNA: Deoxyribonucleic acid
EDTA: Ethylenediaminetetraacetic acid
EIA: Enzyme immunoassay
HFD: High-fat diet
IL-6: Interleukin-6
ITT: Insulin tolerance test
MCP1: Monocyte chemotactic protein-1
MRI: Magnetic resonance imaging
ND: Normal diet
NIH: National Institutes of Health
OGTT: Oral glucose tolerance test
PBS: Phosphate-buffered saline
PCR: Polymerase chain reaction
PGC-1α: Peroxisome proliferator-activated receptor gamma coactivator 1
SEM: Standard error of the mean
STZ: Streptozotocin
TNF-α: Tumor necrosis factor-α
